# Letter on the Use of Veratrum Viride

**Published:** 1857-05

**Authors:** 


ARTICLE III.—*Letter on the Use of Veratrum Viride.* From Dr. HILL, of Hopedale, Ill. March, 1857.

DR. DAVIS:

*Sir*—Having just read a communication in the March number of the *Medical and Surgical Journal*, in relation to the further results of the exhibition of the Veratrum Viride in the treatment of fevers, I would gladly contribute my small experience in the value of the drug in question, at least in conformity to the request of Dr. Dewey, that those knowing anything of the result by actual observation should communicate the same as evidence, if indeed further evidence is required of the effects of the remedy in the treatment of fevers.

I have, in the short time that I have been in the practice, given the veratrum almost indiscriminately in fevers, and have almost, without exception, seen it followed by the very best results; however, I have never noticed any decided inclination to diaphoresis that I attributed to the remedy, as has been spoken of by some.

The first case in which I exhibited the veratrum viride, decided me in its favor; it was a case of remittent fever, of a highly sthenic grade of action and bilious complication.

On the first of June last I was called to attend Miss B———, in this vicinity, who had had chills, followed by the usual febrile stage. On the third day of her illness (the day I was called to see her), the intermission, which should have occurred at 3 P.M. was very slightly marked, in fact almost a continued grade of febrile excitement. The whole surface was hot and dry; countenance very much flushed; great pain in the head with considerable cerebral excitement; tongue red on tip and edges, coated with a whitish fur, inclining to yellow, on the median line and root; more or less nausea; bowels somewhat constipated.

I commenced the treatment of the case by a purgative dose of calomel and ext. colocynth comp. ordering it followed in six hours with sulphate magnes. in case of its not having operated by that time. Gave the effervescing draught every hour, in combination with spirits of nitre and a little tartar emetic, while the febrile stage lasted, fully expecting by this means to produce a complete intermission in a few hours: likewise leaving quinia sulphas. to be given at short intervals from the time that the intermission should occur.

Visited the patient next day. The cathartic had operated well; the febrifuge had been given, but no intermission. Found on inquiry that the quinine, either from carelessness of the directions or downright ignorance, had been given—and now, indeed, fever wild and high coursed through every vein. I do assure you my alarm was very great. Constant delirium was present, and the patient presented every feature of active cerebral congestion. To obviate the most pressing feature of the case, the cold douch was resorted to with temporary benefit: but this was only palliative, it was not a cure. I wanted an agent to reduce the force and volume of the circulation, and that, too, promptly. I gave five drops of the tincture verat. viride (of Dr. Norwood), and in half an hour five more, which produced emesis. A rapid diminution in the force and frequency of the pulse was now perceptible ; the delirium was almost entirely relieved; a very decided drowsiness seemed to steal over the patient. I now ordered three drops to be given with a little spirits of nitre every hour and a half, till a complete intermission should occur, intending to visit her in a few hours.

Next morning visited the patient early. Found her sitting up in bed; pulse soft and moderately full, and about the healthy standard in frequency; skin cool and slightly moist; feeling quite comfortable, as she expressed it: in short, almost a complete intermission. Anti-periodic doses of quinine were now administered, and the patient soon recovered.

Here was a case where febrifuge medicines of a diaphoretic and sedative character, failed in producing an intermission sufficiently marked for the exhibition of the sulphate of quinine to cut short the approach of the next paroxysm; but, on the contrary, the fever continued with unabated violence, and it was not until the verat. viride was given at first in large, and next in generally decreasing doses, that the ultimatum so desired was attained.

I have since given the veratrum in almost every case occuring in my practice, where a control over the force, frequency, and volume of the circulation was principally indicated; and to a considerable extent, at least, has been followed by the expected effect. I sometimes exhibit it after your formula; at other times in a little sweetened water, or elm water, or spirits of nitre alone; generally in larger doses at first, then at more lengthened intervals smaller doses. I have seen it followed by alarming depression, requiring a prompt recourse to alcoholic or opiate stimulus to prevent the apparent syncope to which the patient was hastening.

If you deem this communication of sufficient importance to entitle it publication, it is at your service. The fact of its being the first article that I have ever written for publication, will, I hope, be a sufficient apology for its many imperfections in composition and penmanship, likewise for the intrusion on your time and patience.

